# The association between hematological inflammatory markers and atrial fibrillation recurrence after radiofrequency ablation

**DOI:** 10.3389/fmed.2026.1802752

**Published:** 2026-03-31

**Authors:** Xinxin Su, Xingye Chen, Xiaoyan Wang, Hao Su, Liwu Xu

**Affiliations:** 1 The First Hospital of Anhui University of Science and Technology, Huainan, Anhui, China; 2 Wannan Medical College, Wuhu, Anhui, China; 3 Bengbu Medical University, Bengbu, Anhui, China; 4 Division of Life Sciences and Medicine, Department of Cardiology, First Affiliated Hospital of USTC, University of Science and Technology of China, Hefei, Anhui, China

**Keywords:** atrial fibrillation, atrial fibrillation recurrence, hematological inflammatory markers, inflammation, radiofrequency ablation

## Abstract

**Objective:**

To investigate the relationship between hematological inflammatory markers and postoperative recurrence in patients undergoing initial radiofrequency ablation for atrial fibrillation (AF).

**Methods:**

Patients with AF who underwent their first radiofrequency ablation at a hospital in China were enrolled, and comprehensive clinical data were collected. Multivariable logistic regression analysis assessed the relationship between inflammatory markers and AF recurrence risk. Restricted cubic spline (RCS) analysis was performed to examine potential linear trends. Subgroup analyses evaluated the consistency of results across patient categories. Receiver operating characteristic (ROC) curve analysis compared the discriminatory performance of different inflammatory markers for AF recurrence.

**Results:**

A total of 1,217 patients were included, of whom 236 experienced AF recurrence within 1 year (recurrence rate: 19.4%). In the fully adjusted model, higher NLR (OR = 1.13, 95% CI: 1.03–1.23; *p* = 0.007), NAR (OR = 1.35, 95% CI: 1.05–1.76; *p* = 0.025), SIRI (OR = 1.20, 95% CI: 1.07–1.35; *p* = 0.003), and MLR (OR = 4.54, 95% CI: 1.79–9.55; *p* = 0.002) were independently associated with higher odds of AF recurrence. RCS analysis demonstrated a linear relationship for these markers. Subgroup analysis revealed no significant interaction among different stratification variables. ROC analyses showed limited discriminative performance for AF recurrence (AUCs 0.519–0.554), with MLR having the numerically highest AUC but remaining close to chance-level discrimination.

**Conclusion:**

Elevated levels of the inflammatory markers NLR, NAR, SIRI, and MLR are significantly associated with increased AF recurrence risk following initial radiofrequency ablation. Pre-ablation inflammatory indices should be interpreted as adjunctive markers associated with recurrence, and their potential utility may lie in risk stratification when integrated with established clinical predictors, rather than as standalone predictors.

## 1 Introduction

Atrial fibrillation (AF) is the most common sustained cardiac arrhythmia and is associated with increased risks of stroke, heart failure, cognitive decline, and mortality, substantially impairing quality of life (1–4). With population aging, AF prevalence continues to rise, and an estimated 330 million people are affected worldwide (5, 6).

Treatment for AF is essential to improve prognosis and reduce complications. Management includes drug therapy (anti-arrhythmic and anticoagulant treatment) to control rate, maintain sinus rhythm, and prevent thrombosis (7–10), and interventional options such as radiofrequency ablation, left atrial appendage closure, and pacemaker implantation for patients in whom drug therapy is ineffective or unsuitable (11–15). Radiofrequency ablation is considered the most effective approach (>90% success) with low procedural risk, rapid recovery, and low recurrence (16–19). Recurrence is typically defined as any documented atrial tachyarrhythmia episode (AF, atrial flutter, or atrial tachycardia) lasting >30 s after ablation, detected by electrocardiography or ambulatory monitoring (20). Although long-term follow-up suggests many patients remain free from recurrence, a substantial proportion experience recurrent arrhythmia after a single procedure, and repeat ablation is sometimes required (21, 22). Therefore, early identification and risk stratification of patients prone to recurrence after initial ablation is clinically important.

At present, no single indicator has been clearly established to reliably discriminate AF recurrence after ablation. Inflammation is considered closely involved in atrial structural and electrical remodeling, and AF itself may further amplify inflammatory activation, forming a self-perpetuating cycle (23). Prior studies have shown that elevated circulating inflammatory factors are associated with AF recurrence after cardiac surgery and following radiofrequency ablation, supporting an inflammatory contribution to recurrent arrhythmia (24).

Recently, several complete blood count (CBC)-derived inflammatory indices have gained attention, including NAR, NLR, PLR, SIRI, AISI, and SII (25–27). These indices are inexpensive, widely available, and reproducible, and may reflect the balance between inflammatory activation and immune regulation (25–27). Existing evidence suggests that higher levels of these markers are associated with AF and with recurrence risk after ablation, potentially through neutrophil−/monocyte-related oxidative stress and cytokine release driving remodeling, while lymphopenia may indicate impaired anti-inflammatory regulation (28, 29). However, the clinical utility and discriminatory performance of these CBC-derived indices for post-ablation recurrence remain insufficiently clarified.

Therefore, this study aimed to evaluate the associations between multiple hematological inflammatory indices and AF recurrence after initial radiofrequency ablation, and to assess their discriminatory performance for identifying patients at higher risk of recurrence. The results may support earlier risk stratification and more individualized post-ablation management.

## 2 Methods

### 2.1 Study design and population

**Figure 1:**
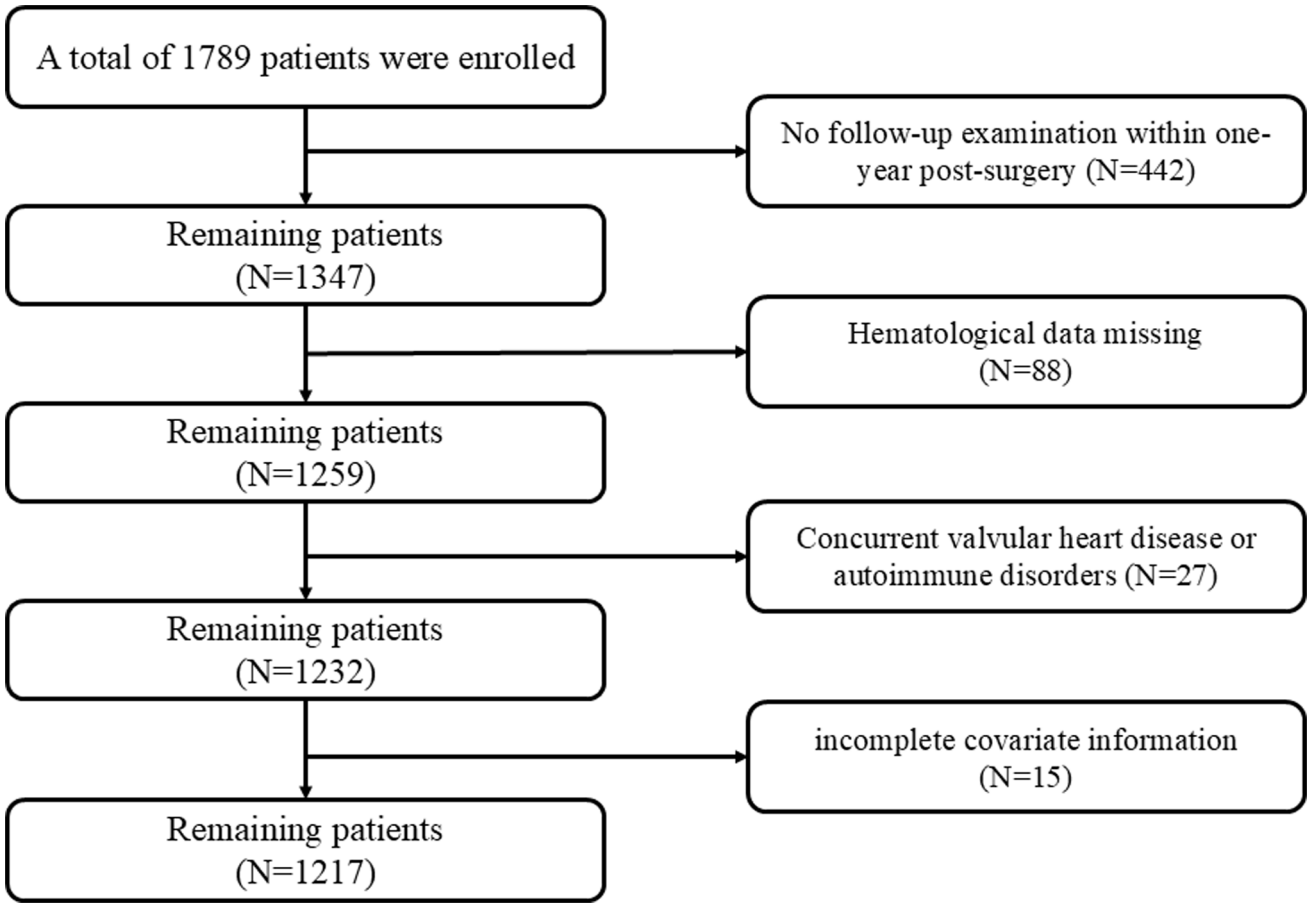
Flowchart for selecting patients. This flowchart summarizes the screening and selection of patients undergoing initial radiofrequency ablation for atrial fibrillation (AF). A total of 1789 patients were initially assessed. Exclusions included no follow-up examination within 1 year after surgery (*n* = 442), missing hematological data (*n* = 88), concurrent valvular heart disease or autoimmune disorders (*n* = 27), and incomplete covariate information (*n* = 15). After applying these criteria, 1,217 patients were included in the final analysis. Notably, loss to 1-year follow-up was the main reason for exclusion. AF, atrial fibrillation.

This study was a single-center retrospective cohort study. It included patients who underwent their first radiofrequency ablation for AF at the Department of Cardiology, First Affiliated Hospital of the University of Science and Technology of China (USTC), between January 1 and December 31, 2023. The exclusion criteria were: (1) lack of evidence for AF recurrence (no follow-up within 1 year after the procedure); (2) absence of hematological test data; (3) history of valvular heart disease or autoimmune disease; and (4) missing covariate information. The study flow is shown in Figure 1. Information on chronic anti-inflammatory medication use and thyroid function status was not consistently available in this retrospective database and thus was not used as exclusion criteria or adjustment variables. Ultimately, 1,217 eligible patients were enrolled. This retrospective study was conducted in accordance with the Declaration of Helsinki and was approved by the Ethics Committee of the First Affiliated Hospital of University of Science and Technology of China (Ethics Committee No. 2024-RE-399). Because of the retrospective observational design and use of de-identified clinical data, the requirement for written informed consent was waived by the Ethics Committee.

### 2.2 Evaluation of recurrent AF events

AF recurrence was assessed through intermittent rhythm monitoring, including scheduled outpatient visits with 12-lead electrocardiography and symptom-triggered recordings, supplemented by 24-h Holter monitoring when clinically indicated. Because continuous monitoring (e.g., implantable loop recorders) was not routinely available, asymptomatic or brief paroxysmal episodes may have been missed. Recurrent AF events were assessed through the outpatient electronic medical record system. Patients were evaluated based on clinical diagnosis or electrocardiogram results at outpatient visits 1, 3, 6, 9, and 12 months after the procedure. As a result, the observed 1-year recurrence rate in this study may be underestimated, particularly for asymptomatic or short paroxysmal episodes that are less likely to be captured by intermittent monitoring.

### 2.3 Hematological inflammation markers

Hematological test results were collected from routine pre-ablation blood tests. Blood samples were obtained after at least 8 h of fasting and analyzed following standard operating procedures in the hospital’s clinical laboratory. All inflammatory indices were calculated from a single measurement obtained 1–3 days before the ablation procedure after hospital admission. The inflammatory markers were calculated using the following formulas:

- NAR = neutrophil count/albumin
- NLR = neutrophil count/lymphocyte count
- PLR = platelet count/lymphocyte count
- MLR = monocyte count/lymphocyte count
- Systemic Inflammatory Response Index (SIRI) = (neutrophils × monocytes) /lymphocytes
- Aggregate Systemic Inflammatory Index (AISI) = (platelets × neutrophils × monocytes) /lymphocytes
- Systemic Immunological Inflammation Index (SII) = (platelets × neutrophils)/ lymphocytes.

### 2.4 Covariates

Demographic variables included age and sex. Anthropometric data comprised height and weight, measured with participants barefoot and wearing light clothing. Body mass index (BMI) was calculated as weight (kg) divided by height squared (m^2^). Blood pressure was measured after a five-minute rest using an automated sphygmomanometer. Hypertension was defined as systolic pressure ≥140 mmHg or diastolic pressure ≥90 mmHg. Laboratory assessments included alanine aminotransferase (ALT), aspartate aminotransferase (AST), and fasting blood glucose (FBG). ALT and AST were used to evaluate hepatic function. Diabetes was diagnosed based on a self-reported history, hypoglycemic medication use, or an FBG level ≥7.0 mmol/L. Lifestyle information was obtained using standardized questionnaires. Smoking status was categorized as “smoker” or “non-smoker,” and alcohol intake was classified as “<1 drink/week,” “1–2 drinks/week,” or “>2 drinks/week.”

### 2.5 Data analysis

Participants were divided into recurrent and non-recurrent AF groups, and baseline characteristics were compared. Continuous variables were presented as mean ± standard deviation (SD) and compared using the rank-sum test. Categorical variables were expressed as counts (percentages) and analyzed using the chi-square test. To examine associations between inflammatory markers and AF recurrence, three multivariable logistic regression models were constructed: Model 1 (unadjusted); Model 2 (adjusted for age and sex); and Model 3 (further adjusted for smoking status, alcohol consumption, hypertension, diabetes, BMI, ALT, and AST). RCS curves were used to explore potential linear or nonlinear associations between inflammatory markers and AF recurrence. Subgroup analyses were performed by age, sex, BMI, smoking status, alcohol intake, hypertension, and diabetes to explore the consistency of the main associations across clinically relevant patient strata. Interaction *p*-values were calculated by including cross-product terms between each inflammatory marker and the subgroup variable in the corresponding regression model. These subgroup analyses were considered exploratory. Because multiple subgroup comparisons were performed, no formal adjustment for multiple testing was applied, and the interaction results should therefore be interpreted cautiously. In addition, the study was not specifically powered to detect subgroup-by-marker interaction effects; thus, the absence of statistically significant interaction should not be interpreted as definitive evidence of no effect modification. ROC analyses were used to compare the standalone discriminatory performance of individual inflammatory markers for AF recurrence rather than to develop a comprehensive clinical prediction model. Because ROC analyses suggested overall limited discrimination and MLR had the numerically highest AUC among the tested single markers, we performed 10-fold cross-validation for MLR as a limited internal validation of the most promising individual marker. This validation was not applied to all inflammatory markers and should not be interpreted as a comprehensive validation of a broader predictive-modeling framework. The mean cross-validated AUC and its 95% CI are reported in Supplementary Figure S1. Inflammatory indices were analyzed as continuous variables on their original scales (i.e., not z-standardized and not log-transformed) in the primary analyses. Accordingly, odds ratios (ORs) from logistic regression represent the change in odds of AF recurrence per 1-unit increase in each index. Because the numeric ranges differ across indices, OR magnitudes should not be directly compared across markers. To minimize multicollinearity arising from shared blood cell components, each inflammatory index was entered into separate multivariable models. We additionally assessed collinearity using variance inflation factors (VIFs); all VIFs were < 5 (Supplementary Table S1).

### 2.6 Covariate selection

Covariates were selected *a priori* based on clinical knowledge and prior evidence as potential confounders that may be associated with both systemic inflammation and AF recurrence after ablation, and according to availability and completeness in this retrospective dataset. We adopted a stepwise adjustment strategy to assess the robustness of associations: Model 1 was unadjusted; Model 2 adjusted for key demographic and anthropometric factors (age, sex, and BMI); and Model 3 further adjusted for lifestyle factors (smoking and drinking) and major cardiometabolic comorbidities (hypertension and diabetes), which may influence inflammatory status and recurrence risk. ALT and AST were additionally included as markers of hepatic/metabolic status that may correlate with systemic inflammation. Because CBC-derived indices share overlapping components, each inflammatory index was evaluated in separate models to avoid multicollinearity.

### 2.7 Ablation procedure

All catheter ablation procedures were performed at the First Affiliated Hospital of USTC by experienced electrophysiologists using a standardized institutional protocol. Radiofrequency ablation was performed under local anesthesia with conscious sedation, according to routine institutional practice. Pulmonary vein isolation (PVI) was performed in all patients. Additional ablation (e.g., linear lesions or complex fractionated atrial electrograms) was performed at the operator’s discretion according to the standardized workflow and patient-specific substrate. Periprocedural anticoagulation and antiarrhythmic drug management followed guideline-recommended practice.

The procedures were performed by multiple operators. When multiple operators were involved, the protocol, and endpoint definitions were standardized across operators.

## 3 Results

### 3.1 Baseline characteristics of patients in both groups

Patients were categorized as having experienced or not experienced AF recurrence within 1 year after radiofrequency ablation. Among the 1,217 participants, 737 (60.56%) were men, with a mean age of 65.12 years. A total of 236 patients experienced AF recurrence, resulting in a recurrence rate of 19.4%. Detailed baseline characteristics are shown in Table 1. Higher levels of NAR, NLR, SIRI, and MLR were observed in the AF recurrence group than in the non-recurrence group.

**Table 1:** Demographics classified with recurrence of atrial fibrillation.

Variable	All	Non-recurrence of AF	Recurrence of AF	*p*-value
Number	1,217	981	236	
Gender (*N*, %)				0.305
Male	737 (60.56)	601 (61.26)	136 (57.63)	
Female	480 (39.44)	380 (38.74)	100 (42.37)	
AGE, Mean ± SD	65.12 ± 10.53	64.68 ± 10.48	66.95 ± 10.57	0.003
BMI (N, %)				0.643
<25 kg/m^2^	634 (52.10)	507 (51.68)	127 (53.81)	
(25, 30) kg/m^2^	489 (40.18)	395 (40.27)	94 (39.83)	
>30 kg/m^2^	94 (7.72)	79 (8.05)	15 (6.36)	
Drink (N, %)				0.145
<1 cup /week	817 (67.13)	659 (67.18)	158 (66.95)	
1-2cups /week	59 (4.85)	53 (5.40)	6 (2.54)	
>2cups /week	341 (28.02)	269 (27.42)	72 (30.51)	
Smoking status (*N*, %)				0.045
Yes	132 (10.85)	115 (11.72)	17 (7.20)	
No	1,085 (89.15)	866 (88.28)	219 (92.80)	
Hypertension (*N*, %)				0.018
Yes	404 (33.20)	341 (34.76)	63 (26.69)	
No	813 (66.80)	640 (65.24)	173 (73.31)	
Diabetes (*N*, %)				0.620
Yes	128 (10.60)	101 (10.38)	27 (11.49)	
No	1,080 (89.40)	872 (89.62)	208 (88.51)	
AST, Mean ± SD	22.53 ± 17.97	22.53 ± 19.17	22.50 ± 11.80	0.983
ALT, Mean ± SD	23.97 ± 21.96	24.42 ± 22.13	22.10 ± 21.20	0.146
TG, Mean ± SD	1.48 ± 1.00	1.50 ± 1.04	1.41 ± 0.82	0.218
NAR, Mean ± SD	0.08 ± 0.04	0.08 ± 0.04	0.09 ± 0.04	0.030
NLR, Mean ± SD	2.31 ± 1.55	2.24 ± 1.31	2.59 ± 2.27	0.022
SIRI, Mean ± SD	1.06 ± 1.15	1.00 ± 0.84	1.30 ± 1.97	0.028
AISI, Mean ± SD	188.00 ± 196.87	180.90 ± 161.04	217.50 ± 302.18	0.073
MLR, Mean ± SD	0.27 ± 0.14	0.26 ± 0.12	0.30 ± 0.21	0.004
PLR, Mean ± SD	110.57 ± 46.37	109.81 ± 46.39	113.71 ± 46.24	0.246
SII, Mean ± SD	405.13 ± 278.84	398.61 ± 256.25	432.22 ± 357.21	0.096

This table presents the baseline demographic characteristics of all patients, as well as comparisons between those with and without AF recurrence after initial radiofrequency ablation. Continuous variables are reported as means ± standard deviation (SD), and categorical variables are presented as counts and percentages. The *p*-value for each variable is included to assess differences between patients with AF recurrence and those without.

AF, atrial fibrillation; BMI, body mass index; SD, standard deviation; NLR, neutrophil count/lymphocyte count; NAR, neutrophil-to-albumin ratio; SIRI, systemic inflammation response index; MLR, monocyte-to-lymphocyte ratio; TG, triglycerides; ALT, alanine aminotransferase; AST, aspartate aminotransferase.

Overall, age (but not gender) differed between groups, and several CBC-derived inflammatory indices were higher in the recurrence group.

### 3.2 Multivariate logistic regression and RCS

Multivariate logistic regression was used to analyze the association between inflammatory markers and AF recurrence. In the fully adjusted model (Model 3), NAR [OR (95% CI): 1.35 (1.05–1.76), *p* = 0.025], NLR [OR (95% CI): 1.13 (1.03–1.23), *p* = 0.007], SIRI [OR (95% CI): 1.20 (1.07–1.35), *p* = 0.003], and MLR [OR (95% CI): 4.54 (1.79–9.55), *p* = 0.002] were positively associated with AF recurrence. These ORs are expressed per 1-unit increase in each index on its original scale; therefore, the larger OR observed for MLR should be interpreted in the context of its smaller numeric range and should not be directly compared with ORs of other indices. No association was found between AISI, PLR, or SII and AF recurrence. To further clarify these associations, inflammatory markers were evaluated using quartile analysis (Table 2).

**Table 2:** The relationship between hematological inflammatory markers and incidence of recurrence of atrial fibrillation.

Variable	Model 1		Model 2		Model 3	
	OR (95% CI)	*p-*value	OR (95% CI)	*p-*value	OR (95% CI)	*p-*value
NAR (continuous)	**1.37 (1.07 ~ 1.76)**	0.013	1.36 (1.06 ~ 1.75)	0.017	**1.35 (1.05 ~ 1.76)**	**0.025**
NAR (multi-category)						
Q1	ref	ref	ref	ref	ref	ref
Q2	1.00 (0.66 ~ 1.52)	1.000	1.00 (0.65 ~ 1.52)	0.986	1.00 (0.66 ~ 1.53)	0.989
Q3	1.24 (0.83 ~ 1.86)	0.303	1.26 (0.84 ~ 1.90)	0.272	1.28 (0.85 ~ 1.94)	0.242
Q4	1.33 (0.89 ~ 1.99)	0.160	1.32 (0.88 ~ 1.98)	0.181	1.34 (0.88 ~ 2.04)	0.166
The relationship between NLR and incidence of recurrence of atrial fibrillation						
NLR (continuous)	1.13 (1.04 ~ 1.23)	0.003	1.12 (1.03 ~ 1.21)	0.008	**1.13 (1.03 ~ 1.23)**	**0.007**
NLR (multi-category)						
Q1	ref	ref	ref	ref	ref	ref
Q2	1.05 (0.68 ~ 1.60)	0.828	1.04 (0.68 ~ 1.60)	0.859	1.02 (0.66 ~ 1.57)	0.937
Q3	1.35 (0.90 ~ 2.04)	0.147	1.32 (0.87 ~ 1.99)	0.189	1.28 (0.84 ~ 1.95)	0.244
Q4	1.51 (1.01 ~ 2.27)	0.045	1.41 (0.93 ~ 2.13)	0.108	1.35 (0.89 ~ 2.06)	0.162
The relationship between SIRI and incidence of recurrence of atrial fibrillation						
SIRI (continuous)	1.19 (1.07 ~ 1.34)	0.002	1.18 (1.06 ~ 1.33)	0.004	**1.20 (1.07 ~ 1.35)**	**0.003**
SIRI (multi-category)						
Q1	ref	ref	ref	ref	ref	ref
Q2	1.53 (1.01 ~ 2.34)	0.046	1.54 (1.01 ~ 2.35)	0.046	**1.55 (1.01 ~ 2.37)**	**0.045**
Q3	1.47 (0.97 ~ 2.25)	0.071	1.50 (0.97 ~ 2.31)	0.066	1.47 (0.95 ~ 2.28)	0.081
Q4	1.56 (1.03 ~ 2.37)	0.038	1.49 (0.97 ~ 2.29)	0.071	1.47 (0.95 ~ 2.27)	0.085
The relationship between AISI and incidence of recurrence of atrial fibrillation						
AISI (continuous)	1.16 (0.96 ~ 1.41)	0.122	1.16 (0.95 ~ 1.41)	0.141	1.16 (0.95 ~ 1.42)	0.144
AISI (multi-category)						
Q1	ref	ref	ref	ref	ref	ref
Q2	1.04 (0.69 ~ 1.57)	0.835	1.06 (0.70 ~ 1.59)	0.787	1.06 (0.70 ~ 1.60)	0.792
Q3	1.09 (0.73 ~ 1.63)	0.680	1.10 (0.73 ~ 1.66)	0.636	1.08 (0.71 ~ 1.63)	0.724
Q4	1.13 (0.76 ~ 1.69)	0.552	1.11 (0.74 ~ 1.66)	0.621	1.10 (0.73 ~ 1.67)	0.640
The relationship between MLR and incidence of recurrence of atrial fibrillation						
MLR (continuous)	5.38 (2.19 ~ 13.17)	<0.001	4.80 (1.89 ~ 9.18)	0.001	**4.54 (1.79 ~ 9.55)**	**0.002**
MLR (multi-category)						
Q1	ref	ref	ref	ref	ref	ref
Q2	1.34 (0.88 ~ 2.05)	0.167	1.32 (0.86 ~ 2.01)	0.207	1.28 (0.83 ~ 1.97)	0.257
Q3	1.32 (0.87 ~ 2.02)	0.194	1.28 (0.84 ~ 1.97)	0.255	1.27 (0.82 ~ 1.96)	0.284
Q4	1.62 (1.08 ~ 2.44)	0.021	1.49 (0.97 ~ 2.30)	0.068	1.41 (0.91 ~ 2.18)	0.124
The relationship between PLR and incidence of recurrence of atrial fibrillation						
PLR (continuous)	1.00 (0.99 ~ 1.00)	0.246	1.00 (0.99 ~ 1.00)	0.351	1.00 (0.99 ~ 1.00)	0.541
PLR (multi-category)						
Q1	ref	ref	ref	ref	ref	ref
Q2	1.32 (0.88 ~ 2.00)	0.179	1.30 (0.87 ~ 1.97)	0.203	1.24 (0.82 ~ 1.88)	0.308
Q3	1.12 (0.74 ~ 1.70)	0.594	1.10 (0.72 ~ 1.66)	0.681	1.00 (0.65 ~ 1.54)	0.996
Q4	1.34 (0.90 ~ 2.02)	0.155	1.29 (0.86 ~ 1.95)	0.215	1.20 (0.79 ~ 1.82)	0.396
The relationship between SII and incidence of recurrence of atrial fibrillation						
SII (continuous)	1.00 (0.99 ~ 1.00)	0.100	1.00 (0.99 ~ 1.00)	0.133	1.00 (0.99 ~ 1.00)	0.156
SII (multi-category)						
Q1	ref	ref	ref	ref	ref	ref
Q2	1.31 (0.86 ~ 1.98)	0.207	1.31 (0.86 ~ 1.99)	0.206	1.30 (0.86 ~ 1.99)	0.217
Q3	1.53 (1.02 ~ 2.30)	0.041	1.54 (1.02 ~ 2.32)	0.039	1.49 (0.99 ~ 2.26)	0.058
Q4	1.20 (0.79 ~ 1.82)	0.403	1.16 (0.76 ~ 1.77)	0.500	1.11 (0.72 ~ 1.71)	0.637

Model 1: unadjusted.

Model 2: Model 1 + gender and age.

Model 3: Model 2 + TG; BMI; Drink; Smoke; Hypertension; ALT; AST and Diabetic.

BMI, body mass index; OR, odds ratio; CI, confidence interval; NAR, Neutrophil-to-albumin ratio; NLR, Neutrophil-to-lymphocyte ratio; SII, Systemic immunological inflammation index; AISI, Aggregate systemic inflammatory index; MLR, Monocyte-to-lymphocyte ratio; PLR, Platelet-to-lymphocyte ratio; TG, Triglycerides; BMI, Body mass index; ALT, Alanine aminotransferase; AST, Aspartate aminotransferase; OR, Odds ratio; CI, Confidence interval; SD, Standard deviation.

This table shows the association between various inflammatory markers (NAR, NLR, SIRI, AISI, MLR, PLR) and the incidence of atrial fibrillation recurrence after initial radiofrequency ablation. The results are presented for three models: Model 1 (unadjusted), Model 2 (adjusted for gender and age), and Model 3 (adjusted for TG, BMI, drinking, smoking, hypertension, ALT, AST, and diabetes). The odds ratio (OR) and 95% confidence interval (95% CI) are reported, along with the P-value for each variable.

For continuous analyses, ORs represent the change in odds of AF recurrence per 1-unit increase in each inflammatory index on its original scale (no z-standardization or log transformation). Because indices have different numeric ranges, OR magnitudes are not directly comparable across markers. After full adjustment (Model 3), higher NLR (continuous), NAR (continuous), SIRI (continuous), and MLR (continuous) were independently associated with increased odds of AF recurrence (all *p* < 0.05).

**Figure 2:**
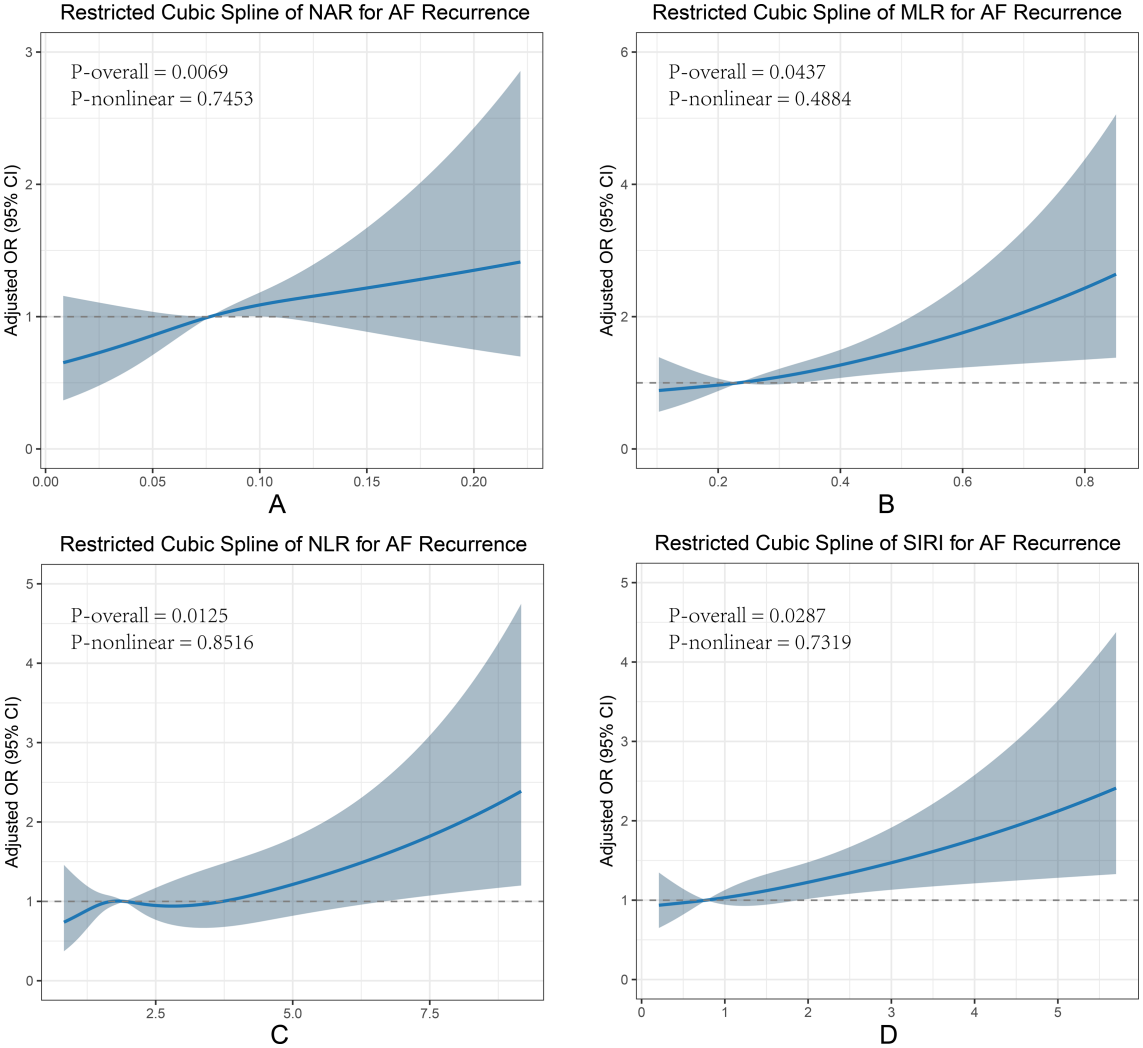
RCS curve for inflammatory markers and AF recurrence. Panels show restricted cubic spline curves for **(A)** neutrophil-to-albumin ratio (NAR), **(B)** monocyte-to-lymphocyte ratio (MLR), **(C)** neutrophil-to-lymphocyte ratio (NLR), and **(D)** systemic inflammation response index (SIRI) measured 1–3 days before ablation. The x-axis represents the value of each inflammatory index, and the y-axis represents the adjusted odds ratio (OR) for AF recurrence with 95% confidence intervals (CIs). The solid line indicates the adjusted OR estimated from the spline model, the shaded band indicates the 95% CI, and the horizontal dashed line denotes OR = 1 (no association). P-overall tests the overall association of each index with recurrence, whereas P-nonlinear tests deviation from linearity (P-nonlinear > 0.05 indicates no evidence of non-linear association). Models were adjusted for age, sex, body mass index (BMI), smoking, drinking, hypertension, diabetes, alanine aminotransferase (ALT), and aspartate aminotransferase (AST). Across all four indices, P-overall was < 0.05 and P-nonlinear was > 0.05, suggesting approximately linear positive associations with recurrence risk within the observed ranges. AF, atrial fibrillation; RCS, restricted cubic spline; OR, odds ratio; CI, confidence interval; NAR, neutrophil-to-albumin ratio; MLR, monocyte-to-lymphocyte ratio; NLR, neutrophil-to-lymphocyte ratio; SIRI, systemic inflammation response index; BMI, body mass index; ALT, alanine aminotransferase; AST, aspartate aminotransferase.

RCS analysis was then performed to examine the linear association between inflammatory biomarkers and AF recurrence (Figure 2). After adjustment for all covariates, positive linear correlations were observed for NAR, NLR, MLR, and SIRI (P-nonlinear > 0.05 for all).

### 3.3 Subgroup analysis

**Figure 3:**
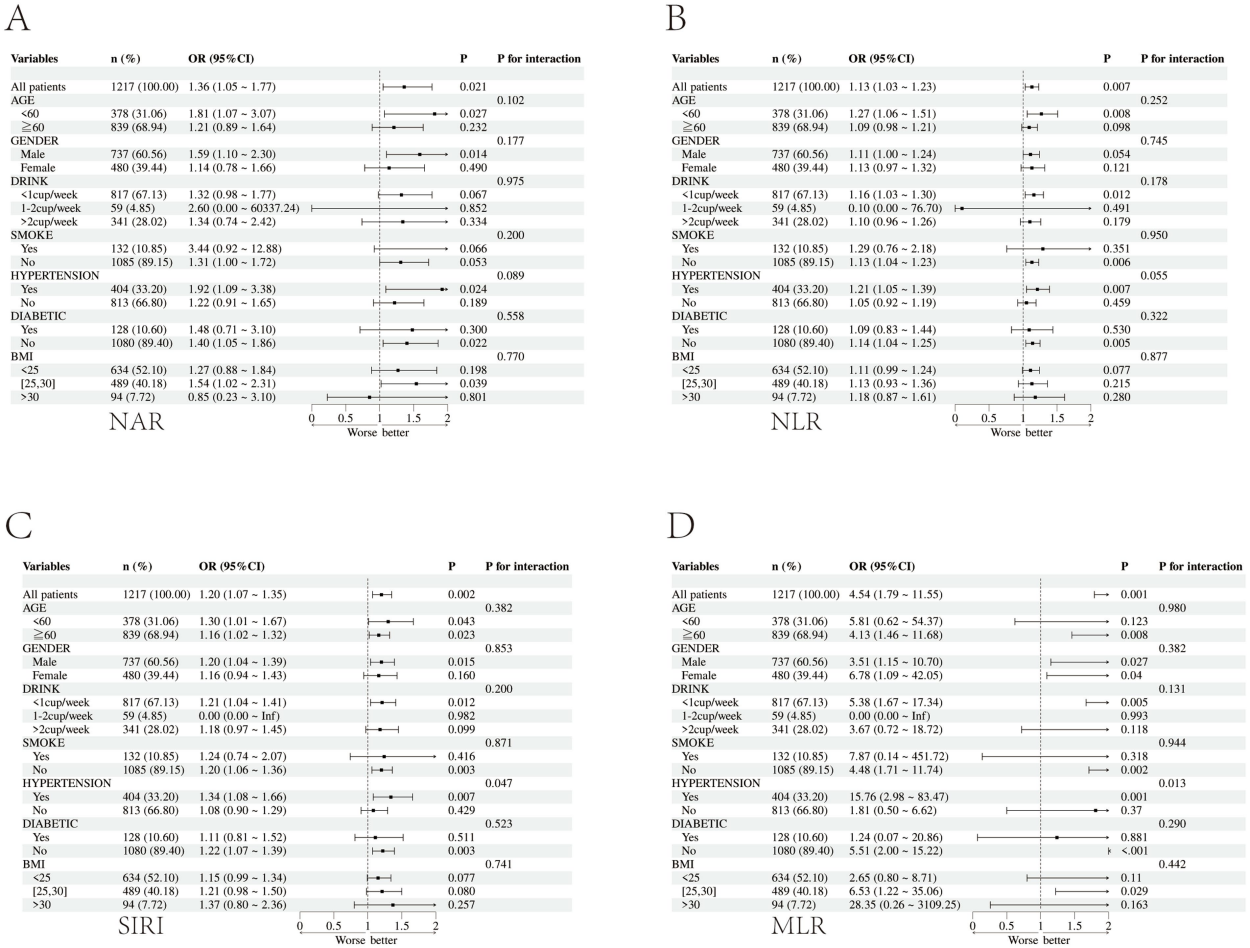
Relationship between inflammatory markers in subgroups and AF recurrence. Forest plots display adjusted odds ratios (ORs) and 95% confidence intervals (CIs) for AF recurrence associated with higher levels of each inflammatory index measured 1–3 days before ablation: **(A)** Neutrophil-to-albumin ratio (NAR), **(B)** neutrophil-to-lymphocyte ratio (NLR), **(C)** systemic inflammation response index (SIRI), and **(D)** monocyte-to-lymphocyte ratio (MLR). For each prespecified subgroup (age, sex, drinking status, smoking status, hypertension, diabetes, and body mass index [BMI] categories), the square marker indicates the subgroup-specific OR and the horizontal line indicates the 95% CI; the vertical reference line denotes OR = 1 (no association). The *x*-axis is plotted on a logarithmic scale for ORs. *P* for interaction tests whether the association between the inflammatory index and recurrence differs across subgroup strata; models were adjusted for age, sex, BMI, smoking, drinking, hypertension, diabetes, ALT, and AST, excluding the stratification variable when applicable. Associations appeared broadly consistent across subgroups, and no statistically significant interaction was detected (all *P* for interaction > 0.05). However, these exploratory subgroup analyses were not adjusted for multiple comparisons and may have been underpowered to detect modest effect modification. AF, atrial fibrillation; OR, odds ratio; CI, confidence interval; NAR, neutrophil-to-albumin ratio; NLR, neutrophil-to-lymphocyte ratio; SIRI, systemic inflammation response index; MLR, monocyte-to-lymphocyte ratio; BMI, body mass index; ALT, alanine aminotransferase; AST, aspartate aminotransferase.

To assess whether the associations between inflammatory markers and AF recurrence differed across patient strata, patients were stratified by age, sex, BMI, smoking status, alcohol consumption, hypertension, and diabetes, and interaction *p*-values were calculated (Figure 3). No statistically significant interactions were detected (all P for interaction > 0.05). However, these subgroup analyses were exploratory and should be interpreted cautiously because multiple subgroup comparisons were performed and the study was not specifically powered to detect modest interaction effects.

### 3.4 ROC curve analysis

**Figure 4:**
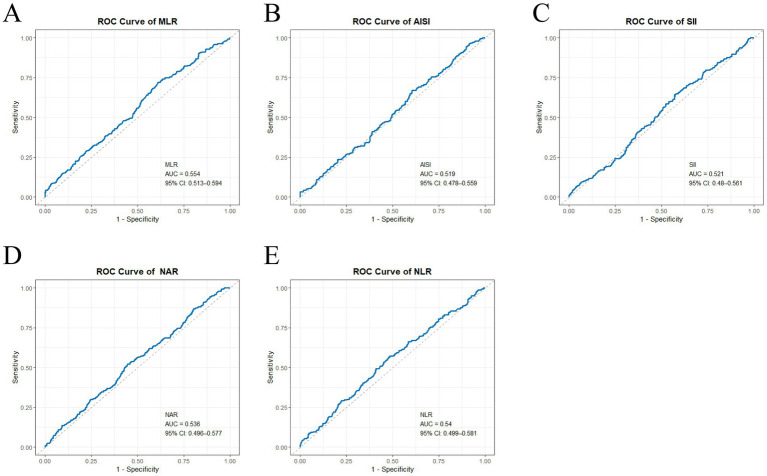
ROC curves of MLR, NAR, NLR, SII, and AISI for discriminating AF recurrence. ROC curves are shown for **(A)** monocyte-to-lymphocyte ratio (MLR), **(B)** aggregate systemic inflammatory index (AISI), **(C)** systemic immunological inflammation index (SII), **(D)** neutrophil-to-albumin ratio (NAR), and **(E)** neutrophil-to-lymphocyte ratio (NLR). The *x*-axis denotes 1 − specificity (false-positive rate) and the *y*-axis denotes sensitivity (true-positive rate). The solid line represents the ROC curve for each index, and the dashed diagonal indicates no discrimination (AUC = 0.50). The area under the curve (AUC) with 95% confidence interval (CI) is reported within each panel. Overall, all indices demonstrate modest standalone discrimination for AF recurrence, with AUCs close to 0.50. The ROC curves largely overlap and differences between indices are small; although MLR has the numerically highest AUC, these markers should be interpreted as adjunctive risk markers rather than standalone predictors. AF, atrial fibrillation; ROC, receiver operating characteristic; AUC, area under the curve; NAR, neutrophil-to-albumin ratio; NLR, neutrophil-to-lymphocyte ratio; MLR, monocyte-to-lymphocyte ratio; SII, systemic immune-inflammation index; AISI, aggregate index of systemic inflammation.

ROC curve analysis demonstrated the discriminatory performance of the inflammatory markers for AF recurrence (Figure 4). In the ROC analysis, MLR (AUC = 0.554, 95% CI: 0.513–0.594), NLR (AUC = 0.540, 95% CI: 0.499–0.581), NAR (AUC = 0.536, 95% CI: 0.496–0.577), SII (AUC = 0.521, 95% CI: 0.480–0.561), and AISI (AUC = 0.519, 95% CI: 0.478–0.559) showed limited discriminative ability for AF recurrence (all AUCs close to 0.50), suggesting that these inflammatory indices have overall weak performance when used as single markers. Overall, the AUCs ranged from 0.519 to 0.554, indicating limited discriminative ability. Although MLR had the numerically highest AUC, the absolute discrimination was modest and close to 0.50, indicating weak standalone performance. In an internal 10-fold cross-validation of MLR, selected because it had the numerically highest observed AUC among the tested single markers, the mean AUC remained 0.55 (95% CI: 0.509–0.591), consistent with limited standalone discrimination. This cross-validation was intended as a limited internal validation of the most promising individual marker and was not performed for all inflammatory indices (Supplementary Figure S1).

## 4 Discussion

In this study, the association between inflammatory markers and recurrence of AF after initial radiofrequency ablation was investigated. The results showed a positive linear correlation between AF recurrence and levels of NLR, NAR, SIRI, and MLR. Subgroup analyses showed no clear statistical evidence of effect modification (all P for interaction > 0.05); however, these exploratory analyses involved multiple comparisons and may have been underpowered to detect modest heterogeneity across strata. In addition, MLR showed the highest AUC among the tested indices; however, the overall AUC values were close to 0.50, indicating that these markers provide limited standalone discrimination. We therefore interpreted the ROC analyses as exploratory comparisons of standalone marker discrimination rather than as evidence of a validated predictive framework. The additional cross-validation of MLR was intended only to examine the stability of the highest observed single-marker AUC. The comparatively larger OR observed for MLR likely reflects, at least in part, scale-dependent interpretation, because ORs were estimated per 1-unit increase on the original scale and the distributions/ranges differ across indices. In addition, these CBC-derived indices share overlapping components (neutrophils, lymphocytes, and/or monocytes), implying partial redundancy. We therefore evaluated each index in separate models and confirmed no substantial multicollinearity using VIFs (all < 5; Supplementary Table S1). Several established determinants of post-ablation recurrence—including AF type (paroxysmal vs. persistent), AF duration, left atrial diameter/volume, structural heart disease characteristics, and procedural details (e.g., lesion sets beyond PVI, acute endpoints, operator variability, and peri-procedural antiarrhythmic strategies)—were not available in this retrospective dataset. These factors reflect atrial remodeling and disease severity and may also correlate with systemic inflammation. Therefore, the observed associations between CBC-derived inflammatory indices and recurrence may partly represent unmeasured substrate severity or procedural complexity, and the adjusted ORs may be influenced by residual confounding. Accordingly, these indices should be interpreted as adjunctive correlates rather than standalone substitutes for established clinical, echocardiographic, and procedural predictors.

AF is the most common arrhythmia, increasing the risks of stroke, heart failure, cardiac arrest, and death (30, 31). The development and progression of AF are closely associated with inflammatory responses (32). Inflammation and fibrosis are considered major pathological changes underlying AF (33–35). Previous studies have shown that neutrophils are involved in the acute inflammatory response by eliminating pathogenic bacteria and damaged cells. Recent studies indicate that neutrophils can release neutrophil extracellular traps (NETs), implicated in chronic conditions such as chronic obstructive pulmonary disease and atherosclerosis. Recent *in vitro* and *in vivo* studies demonstrated significantly elevated neutrophil levels in the peripheral blood of AF patients. Meanwhile, NETs induce cardiomyocyte apoptosis and mitochondrial damage (36).

Albumin participates in both acute and chronic inflammatory processes (37). Earlier studies suggested that reduced serum albumin levels correlate with coronary atherosclerosis, leading to increased blood viscosity, endothelial damage, and platelet activation and aggregation (38, 39). This phenomenon is likely attributable to a decline in albumin’s antioxidant, anti-inflammatory, and antiplatelet aggregation properties. Previous studies identified serum albumin levels as an independent risk factor for AF occurrence (40). Chen et al. reported a negative correlation between serum albumin levels and AF incidence (41). Low serum albumin levels reflect malnutrition and often are associated with poor clinical outcomes.

In recent years, the NAR has emerged as a novel inflammatory marker, attracting increasing research attention. Evidence from NHANES-based studies indicates that higher baseline NAR levels significantly correlate with increased risk of all-cause and cardiovascular mortality in the general population (42). In a population-based analysis, NAR was associated with adverse vascular conditions, suggesting potential prognostic relevance in cardiovascular disease (25). Consistent with these findings, this study demonstrated that AF patients with higher baseline NAR levels after initial radiofrequency ablation had a greater likelihood of recurrence. Moreover, NAR positively correlated with AF recurrence rates.

Patients with higher monocyte-to-lymphocyte ratio (MLR) levels had a higher risk of AF recurrence, aligning with previous clinical findings. For instance, one study reported that changes in MLR correlated with early recurrence following pulmonary vein isolation and potentially late recurrence (43). Another study, focusing on monocyte-driven inflammation, identified elevated baseline MLR as an independently associated factor of AF recurrence after catheter ablation (44). Mechanistically, elevated MLR levels might indicate enhanced release of proinflammatory cytokines (IL-6 and TNF-*α*) from activated monocytes. These cytokines trigger inflammatory cascades in cardiomyocytes, promoting atrial structural remodeling and electrical instability, thereby increasing susceptibility to AF recurrence.

Additionally, the systemic inflammatory response index (SIRI), which incorporates neutrophil, monocyte, and lymphocyte counts, provides a comprehensive assessment of systemic inflammatory activity. This study observed a positive correlation between SIRI and AF recurrence, supporting its role as a potential independent correlate. Furthermore, elevated SIRI levels in patients with ST-elevation myocardial infarction (STEMI) following percutaneous coronary intervention (PCI) were independently associated with new-onset AF (NOAF) (45). Similarly, in hypertensive patients undergoing catheter ablation, SIRI exhibited a non-linear positive correlation with AF recurrence (46). In the postoperative setting, a recent CABG cohort also evaluated preoperative SIRI in relation to new-onset postoperative AF, further supporting the link between perioperative inflammatory activation and AF susceptibility, although the observed discriminative performance appeared modest. This highlights that the strength of association and clinical utility of CBC-derived indices may vary across AF-related clinical scenarios (47). The advantage of SIRI lies in its integrative assessment of inflammation, capturing the synergistic effects of multiple cellular inflammatory pathways. These pathways promote oxidative stress and cytokine-mediated injury, ultimately increasing the likelihood of AF recurrence (48). Inflammation is dynamic and may fluctuate during the peri-procedural period. In this study, inflammatory indices were derived from a single pre-ablation measurement obtained 1–3 days before the procedure, which may not fully capture a patient’s stable baseline inflammatory status. Moreover, these indices could be influenced by transient conditions during hospitalization (e.g., acute stress responses, subclinical infection, or other short-term inflammatory triggers). Therefore, our findings should be interpreted as associations based on a single time point, and future prospective studies with serial pre- and post-ablation measurements are warranted to clarify temporal stability and to distinguish baseline inflammatory burden from procedural injury responses or early recurrence-related biology. These hematological inflammatory indices should be viewed as adjunctive biomarkers. Their potential utility may lie in supporting risk stratification when combined with established clinical and echocardiographic predictors, rather than serving as standalone predictors. The positive correlations of MLR, SIRI, and NLR with AF recurrence suggest that systemic inflammation plays a crucial role in AF recurrence. Since these inflammatory markers are derived from routine blood tests, they are simple, cost-effective, and readily applicable in clinical practice. Therefore, they may serve as practical tools for identifying high-risk patients before ablation, monitoring inflammatory status and recurrence trends postoperatively, and guiding early intervention and personalized management strategies.

Although the present study focuses on conventional association analyses, our findings can serve as a foundation for more structured and interpretable modeling in future work. Inspired by recent feature-engineering frameworks that transform complex biological measurements into process-structured features and use explainability to distinguish compositional signals from interaction-driven effects, future studies on AF recurrence may (i) organize hematological inflammatory indices into conceptual feature groups (e.g., neutrophil-dominant, monocyte-driven, and composite systemic inflammation), (ii) quantify redundancy and shared information across correlated markers rather than relying solely on marginal associations, and (iii) integrate inflammatory markers with clinical/procedural/imaging features within unified models, complemented by stability and feature-attribution analyses. Such approaches may improve interpretability and robustness and help clarify why certain markers, e.g., MLR appear relatively stronger despite modest discriminative performance (49).

### 4.1 Limitations and strengths

The strength of this study is its large clinical sample size. In addition, multiple analytical methods were applied, and subgroup analyses were performed, which enhanced the reliability of the findings.

However, this study has several limitations. First, this study was not designed to develop or externally validate a clinical prediction model. In addition, the subgroup analyses should be interpreted with caution. Multiple subgroup and interaction tests were performed without formal adjustment for multiple comparisons, increasing the risk of chance findings. Conversely, with 236 recurrence events, the study was not specifically powered to detect small-to-moderate interaction effects after stratification. Therefore, the absence of statistically significant interactions should not be interpreted as conclusive evidence that the associations are identical across all subgroups. Internal 10-fold cross-validation was performed only for MLR, which had the numerically highest AUC among the tested single markers, and was intended as a limited internal validation of that individual marker. Comparable validation was not performed for all inflammatory indices, nor did we evaluate a broader multivariable predictive-modeling framework. In addition, calibration and clinical utility (e.g., decision-curve analysis) were not assessed, and no external validation was conducted. Accordingly, the ROC/AUC findings should be interpreted as exploratory comparisons of standalone marker discrimination rather than evidence of a mature or clinically applicable prediction model. Therefore, the ROC/AUC results should be interpreted as reflecting limited discriminative performance rather than a mature prediction model. Second, although many confounding factors were adjusted for, the association between inflammatory markers and AF recurrence may still be influenced by unmeasured variables. Third, the observation period was relatively short, as only recurrence within 1 year after the procedure was evaluated. Forth, all patients were recruited from a single region in China, which limits the generalizability of the results to other populations. Further studies are needed to validate these findings in patients with AF in different countries and regions. In addition, inflammatory indices were calculated from a single pre-ablation blood test obtained 1–3 days before the procedure. Given the dynamic nature of inflammation, these values may be affected by transient peri-hospital factors and may not reflect long-term baseline inflammatory status. Serial measurements before and after ablation are needed to improve interpretability and mechanistic inference. Recurrence detection relied primarily on intermittent ECG-based follow-up with selective Holter monitoring rather than continuous rhythm monitoring, which may underestimate recurrence—particularly asymptomatic or short paroxysmal AF episodes. Although we adjusted for several demographic and cardiometabolic covariates, important predictors of recurrence were not captured in this retrospective database, including AF type, AF duration, left atrial diameter/volume, detailed structural heart disease parameters, and granular procedural information (e.g., additional lesion sets, acute isolation durability, operator strategy, and peri-procedural antiarrhythmic management). These variables are related to both atrial substrate severity and recurrence risk and may also be associated with systemic inflammation; thus, residual confounding may remain and our estimates should be interpreted cautiously. In addition, residual confounding may persist due to other unmeasured factors, such as thyroid dysfunction and chronic use of anti-inflammatory medications. Future prospective studies incorporating echocardiographic/imaging, procedural, and rhythm-monitoring data are warranted to assess the incremental value of these indices beyond established predictors. Future prospective studies incorporating echocardiographic/imaging, procedural, and rhythm-monitoring data are warranted to assess the incremental value of these indices beyond established predictors. Importantly, recurrence under-detection would most likely make our estimates conservative, and the true associations may be stronger than observed if missed recurrences are largely non-differential.

## 5 Conclusion

In summary, elevated levels of NLR, NAR, SIRI, and MLR were associated with an increased risk of AF recurrence. Higher pre-ablation inflammatory indices were associated with increased recurrence risk; prospective studies with serial measurements are needed to determine whether peri-procedural inflammation modulation can reduce recurrence. However, additional evidence is needed, and prospective studies are required to confirm this conclusion.

## Data Availability

The raw data supporting the conclusions of this article will be made available by the authors, without undue reservation.

## References

[1] Tan S Zhou J Veang T Lin Q Liu Q. Global, regional, and national burden of atrial fibrillation and atrial flutter from 1990 to 2021: sex differences and global burden projections to 2046-a systematic analysis of the global burden of disease study 2021. Europace. (2025) 27:euaf027. doi: 10.1093/europace/euaf027, 39947238 PMC11879048

[2] Baman JR Passman RS. Atrial fibrillation. JAMA. (2021) 325:2218. doi: 10.1001/jama.2020.23700 34061143

[3] Karakasis P Pamporis K Siontis KC Theofilis P Samaras A Patoulias D . Major clinical outcomes in symptomatic vs. asymptomatic atrial fibrillation: a meta-analysis. Eur Heart J. (2025) 46:1189–202. doi: 10.1093/eurheartj/ehae694, 39428997

[4] Winijkul A Kaewkumdee P Yindeengam A Lip GYH Krittayaphong R. Clinical outcomes of patients with atrial fibrillation who survived from bleeding event: the results from COOL-AF Thailand registry. Thromb Haemost. (2024) 124:991–1002. doi: 10.1055/s-0044-1786028, 38626898

[5] Zakeri R Van Wagoner DR Calkins H Wong T Ross HM Heist EK . The burden of proof: the current state of atrial fibrillation prevention and treatment trials. Heart Rhythm. (2017) 14:763–82. doi: 10.1016/j.hrthm.2017.01.032, 28161513 PMC5403606

[6] Shi S Tang Y Zhao Q Yan H Yu B Zheng Q . Prevalence and risk of atrial fibrillation in China: a national cross-sectional epidemiological study. Lancet Reg Health West Pac. (2022) 23:100439. doi: 10.1016/j.lanwpc.2022.100439, 35800039 PMC9252928

[7] Gutierrez C Blanchard DG. Diagnosis and treatment of atrial fibrillation. Am Fam Physician. (2016) 94:442–52. 27637120

[8] Krepostman N Kramer HJ. Anticoagulation for stroke prevention in atrial fibrillation. Am J Kidney Dis. (2022) 80:561–3. doi: 10.1053/j.ajkd.2022.06.003, 36057469

[9] Markides V Schilling RJ. Atrial fibrillation: classification, pathophysiology, mechanisms and drug treatment. Heart. (2003) 89:939–43. doi: 10.1136/heart.89.8.939, 12860883 PMC1767799

[10] Ravens U Odening KE. Atrial fibrillation: therapeutic potential of atrial K(+) channel blockers. Pharmacol Ther. (2017) 176:13–21. doi: 10.1016/j.pharmthera.2016.10.003, 27742566

[11] Quintanilla JG Shpun S Jalife J Filgueiras-Rama D. Novel approaches to mechanism-based atrial fibrillation ablation. Cardiovasc Res. (2021) 117:1662–81. doi: 10.1093/cvr/cvab108, 33744913 PMC8208747

[12] Rappel WJ Zaman JA Narayan SM. Mechanisms for the termination of atrial fibrillation by localized ablation: computational and clinical studies. Circ Arrhythm Electrophysiol. (2015) 8:1325–33. doi: 10.1161/circep.115.002956, 26359479 PMC4764078

[13] Beigel R Wunderlich NC Ho SY Arsanjani R Siegel RJ. The left atrial appendage: anatomy, function, and noninvasive evaluation. JACC Cardiovasc Imaging. (2014) 7:1251–65. doi: 10.1016/j.jcmg.2014.08.009, 25496544

[14] Saw J Holmes DR Cavalcante JL Freeman JV Goldsweig AM Kavinsky CJ . SCAI/HRS expert consensus statement on transcatheter left atrial appendage closure. Heart Rhythm. (2023) 20:e1–e16. doi: 10.1016/j.hrthm.2023.01.007, 36990925

[15] DeRose JJ Mancini DM Chang HL Argenziano M Dagenais F Ailawadi G . Pacemaker implantation after mitral valve surgery with atrial fibrillation ablation. J Am Coll Cardiol. (2019) 73:2427–35. doi: 10.1016/j.jacc.2019.02.062, 31097163 PMC6602091

[16] Reddy VY Dukkipati SR Neuzil P Anic A Petru J Funasako M . Pulsed field ablation of paroxysmal atrial fibrillation: 1-year outcomes of IMPULSE, PEFCAT, and PEFCAT II. JACC Clin Electrophysiol. (2021) 7:614–27. doi: 10.1016/j.jacep.2021.02.014 33933412

[17] Bianchini L Schiavone M Vettor G Gasperetti A Penza E Ballotta A . Hybrid-convergent procedure or pulsed field ablation in long-standing persistent atrial fibrillation. JACC Clin Electrophysiol. (2024) 10:1700–10. doi: 10.1016/j.jacep.2024.05.029 39084744

[18] January CT Wann LS Calkins H Chen LY Cigarroa JE Cleveland JC . 2019 AHA/ACC/HRS focused update of the 2014 AHA/ACC/HRS guideline for the management of patients with atrial fibrillation: a report of the American College of Cardiology/American Heart Association task force on clinical practice guidelines and the Heart Rhythm Society. J Am Coll Cardiol. (2019) 74:104–32. doi: 10.1016/j.jacc.2019.01.011 30703431

[19] Kautzner J Albenque JP Natale A Maddox W Cuoco F Neuzil P . A novel temperature-controlled radiofrequency catheter ablation system used to treat patients with paroxysmal atrial fibrillation. JACC Clin Electrophysiol. (2021) 7:352–63. doi: 10.1016/j.jacep.2020.11.009, 33516712

[20] Aryana A Chierchia GB de Asmundis C. Recurrent atrial fibrillation after cryoballoon ablation: what to expect! Card Electrophysiol Clin. (2020) 12:199–208. doi: 10.1016/j.ccep.2020.02.002, 32451104

[21] Winkle RA Mead RH Engel G Salcedo J Brodt C Barberini P . Very long term outcomes of atrial fibrillation ablation. Heart Rhythm. (2023) 20:680–8. doi: 10.1016/j.hrthm.2023.02.002, 36764350

[22] Arbelo E Brugada J Hindricks G Maggioni AP Tavazzi L Vardas P . The atrial fibrillation ablation pilot study: a European survey on methodology and results of catheter ablation for atrial fibrillation conducted by the European heart rhythm association. Eur Heart J. (2014) 35:1466–78. doi: 10.1093/eurheartj/ehu001, 24487524

[23] Deftereos SG Beerkens FJ Shah B Giannopoulos G Vrachatis DA Giotaki SG . Colchicine in cardiovascular disease: in-depth review. Circulation. (2022) 145:61–78. doi: 10.1161/circulationaha.121.056171, 34965168 PMC8726640

[24] Luo J Thomassen JQ Nordestgaard BG Tybjærg-Hansen A Frikke-Schmidt R. Neutrophil counts and cardiovascular disease. Eur Heart J. (2023) 44:4953–64. doi: 10.1093/eurheartj/ehad649, 37950632 PMC10719495

[25] Cheng Q Liu C Zhong H Wang Z Zhou S Sun J . Comparison of systemic immunoinflammatory biomarkers for assessing severe abdominal aortic calcification among US adults aged≥40 years: a cross-sectional analysis from NHANES. PLoS One. (2025) 20:e0325949. doi: 10.1371/journal.pone.0325949, 40554552 PMC12186907

[26] Li S Zhang Y Wei W. Association between complete blood count-derived inflammatory biomarkers and renal failure: a cross-sectional study from NHANES 2007-2020. BMJ Open. (2025) 15:e103381. doi: 10.1136/bmjopen-2025-103381, 40889986 PMC12414176

[27] Ha G Yan Z Wu J Wang X Hu J Duan L . Exploring the link between inflammatory biomarkers (SII, SIRI, PLR, NLR, LMR) and migraine in Young and early middle-aged US adults: evidence from NHANES 1999-2004 and machine learning models. Brain Behav. (2025) 15:e70886. doi: 10.1002/brb3.70886, 40977107 PMC12451049

[28] Berezin AE. Predictive value of the systemic immune inflammation index in recurrence of atrial fibrillation after radiofrequency catheter ablation. World J Cardiol. (2025) 17:102981. doi: 10.4330/wjc.v17.i1.102981 39866209 PMC11755125

[29] Jie Q Qian W Jia H Zhang F Wang J. Prognostic value of inflammatory indices for atrial fibrillation recurrence after cryoablation: a cohort study. Front Cardiovasc Med. (2025) 12:1637255. doi: 10.3389/fcvm.2025.1637255, 41040074 PMC12483996

[30] Duytschaever M De Potter T Grimaldi M Anic A Vijgen J Neuzil P . Paroxysmal atrial fibrillation ablation using a novel variable-loop biphasic pulsed field ablation catheter integrated with a 3-dimensional mapping system: 1-year outcomes of the multicenter inspIRE study. Circ Arrhythm Electrophysiol. (2023) 16:e011780. doi: 10.1161/circep.122.011780, 36735937 PMC10026968

[31] Schwennesen HT Andrade JG Wood KA Piccini JP. Ablation to reduce atrial fibrillation burden and improve outcomes: JACC review topic of the week. J Am Coll Cardiol. (2023) 82:1039–50. doi: 10.1016/j.jacc.2023.06.029, 37648353 PMC11103629

[32] Hu YF Chen YJ Lin YJ Chen SA. Inflammation and the pathogenesis of atrial fibrillation. Nat Rev Cardiol. (2015) 12:230–43. doi: 10.1038/nrcardio.2015.2, 25622848

[33] Yang S Mei B Feng K Lin W Chen G Liang M . Long-term results of surgical atrial fibrillation radiofrequency ablation: comparison of two methods. Heart Lung Circ. (2018) 27:621–8. doi: 10.1016/j.hlc.2017.04.016, 28652032

[34] Zhang D Shi J Quan H Liu L Zhang J Guo Y. Five-year results of a modified left atrial maze IV procedure in the treatment of atrial fibrillation: a randomized study. ANZ J Surg. (2020) 90:602–7. doi: 10.1111/ans.15486, 31742849 PMC7217219

[35] Weerasooriya R Khairy P Litalien J Macle L Hocini M Sacher F . Catheter ablation for atrial fibrillation: are results maintained at 5 years of follow-up? J Am Coll Cardiol. (2011) 57:160–6. doi: 10.1016/j.jacc.2010.05.061, 21211687

[36] He L Liu R Yue H Zhang X Pan X Sun Y . Interaction between neutrophil extracellular traps and cardiomyocytes contributes to atrial fibrillation progression. Signal Transduct Target Ther. (2023) 8:279. doi: 10.1038/s41392-023-01497-2, 37491321 PMC10368710

[37] Eckart A Struja T Kutz A Baumgartner A Baumgartner T Zurfluh S . Relationship of nutritional status, inflammation, and serum albumin levels during acute illness: a prospective study. Am J Med. (2020) 133:713–722.e7. doi: 10.1016/j.amjmed.2019.10.031, 31751531

[38] Chien SC Chen CY Lin CF Yeh HI. Critical appraisal of the role of serum albumin in cardiovascular disease. Biomark Res. (2017) 5:31. doi: 10.1186/s40364-017-0111-x, 29152305 PMC5681838

[39] Arques S. Human serum albumin in cardiovascular diseases. Eur J Intern Med. (2018) 52:8–12. doi: 10.1016/j.ejim.2018.04.014, 29680174

[40] Chung MK Martin DO Sprecher D Wazni O Kanderian A Carnes CA . C-reactive protein elevation in patients with atrial arrhythmias: inflammatory mechanisms and persistence of atrial fibrillation. Circulation. (2001) 104:2886–91. doi: 10.1161/hc4901.101760, 11739301

[41] Chen B Wang C Li W. Serum albumin levels and risk of atrial fibrillation: a Mendelian randomization study. Front Cardiovasc Med. (2024) 11:1385223. doi: 10.3389/fcvm.2024.1385223, 38655495 PMC11035896

[42] Han D Wu L Zhou H Li P Liu S Xue Y . Neutrophil percentage-to-albumin ratio and the risk of all-cause and cardiovascular mortality: a 20-year follow-up cohort study of 36,428 US adults. BMC Public Health. (2025) 25:1483. doi: 10.1186/s12889-025-22764-7, 40264041 PMC12013024

[43] Luo Y Zhang J Liu T Yin Z Jin Y Han J . The systemic-immune-inflammation index predicts the recurrence of atrial fibrillation after cryomaze concomitant with mitral valve surgery. BMC Cardiovasc Disord. (2022) 22:45. doi: 10.1186/s12872-022-02494-z, 35152878 PMC8842953

[44] Baba DF Suciu H Avram C Gyorgy M Danilesco A Huma L . Elevated levels of neutrophil-to monocyte ratio are associated with the initiation of paroxysmal documented atrial fibrillation in the first two months after heart transplantation: a Uni-institutional retrospective study. J Cardiovasc Dev Dis. (2023) 10:81. doi: 10.3390/jcdd10020081, 36826577 PMC9960862

[45] Wang J Hu S Liang C Ling Y. The association between systemic inflammatory response index and new-onset atrial fibrillation in patients with ST-elevated myocardial infarction treated with percutaneous coronary intervention. BMC Cardiovasc Disord. (2022) 22:525. doi: 10.1186/s12872-022-02989-9, 36474135 PMC9724303

[46] Zhang Z Li S Tu T Liu C Dai Y Wang C . Nonlinear relationship and predictive value of systemic immune-inflammation index for atrial fibrillation recurrence after catheter ablation in hypertensive patients. Heart Rhythm. (2025) 22:2257–68. doi: 10.1016/j.hrthm.2025.03.1958, 40107395

[47] Erkan MH Rahman OF Durna F. The role of the systemic inflammatory response index in predicting postoperative atrial fibrillation. Rev Assoc Med Bras (1992). (2025) 71:e20240783. doi: 10.1590/1806-9282.20240783, 40172381 PMC11964311

[48] Mangalesh S Dudani S. Systemic inflammatory response index over neutrophil-lymphocyte ratio and monocyte-lymphocyte ratio: comparison of prognostic performance in predicting major adverse cardiac events. Ann Med. (2022) 54:2151–2. doi: 10.1080/07853890.2022.2104919, 35916670 PMC9351579

[49] Tan MJT Kapetanaki M Benos PV. Engineering spatial and molecular features from cellular niches to inform predictions of inflammatory bowel disease. arXiv. (2026). doi: 10.48550/arXiv.2509.09923

