# Supplementary material for: The association between hematological inflammatory markers and atrial fibrillation recurrence after radiofrequency ablation
Source: Front Med (Lausanne). 2026 Mar 31;13:1802752. doi: 10.3389/fmed.2026.1802752 (PMC13076263; doi:10.3389/fmed.2026.1802752)
Supplement: Supplementary file 1 [file Supplementary_file_1.docx]

Supplementary Table S1. VIF values for inflammatory indices (NLR, SII, MLR, NAR, and AISI). All VIFs were <5, indicating no substantial multicollinearity.

| Indicators | VIF |
| --- | --- |
| NLR | 4.168 |
| SII | 4.911 |
| MLR | 3.649 |
| NAR | 1.743 |
| AISI | 4.650 |

NAR = neutrophil count/albumin

NLR = neutrophil count/lymphocyte count

MLR = monocyte count/lymphocyte count

Aggregate Systemic Inflammatory Index (AISI) = (platelets × neutrophils × monocytes) /lymphocytes

Systemic Immunological Inflammation Index (SII) = (platelets × neutrophils)/ lymphocytes.

Supplementary Figure S1. ROC curve of MLR using 10-fold cross-validation for discriminating atrial fibrillation (AF) recurrence.


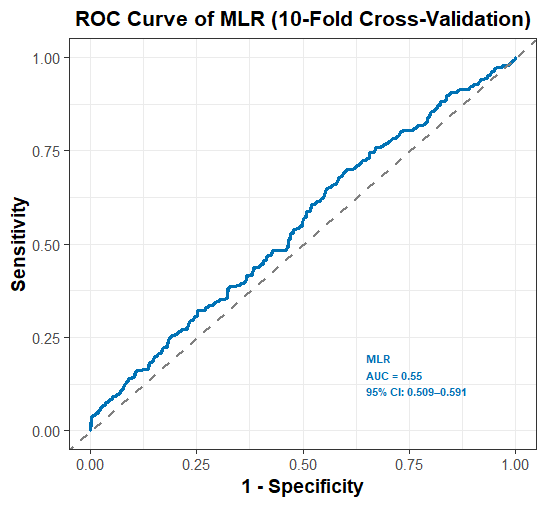


The receiver operating characteristic (ROC) curve evaluates the discriminative performance of the monocyte-to-lymphocyte ratio (MLR). The x-axis represents 1 − specificity (false-positive rate) and the y-axis represents sensitivity (true-positive rate). The solid blue line indicates the ROC curve, and the gray dashed diagonal denotes no-discrimination performance (AUC = 0.50). The cross-validated area under the curve (AUC) was 0.55 with a 95% confidence interval (CI) of 0.509–0.591, indicating limited discriminative ability of MLR as a single marker for AF recurrence.
